# Liquid biopsy for brain metastases and leptomeningeal disease in patients with breast cancer

**DOI:** 10.1038/s41523-023-00550-1

**Published:** 2023-05-24

**Authors:** Stefania Morganti, Heather A. Parsons, Nancy U. Lin, Albert Grinshpun

**Affiliations:** 1 Medical Oncology, Dana-Farber Cancer Institute, Boston, MA USA; 2 Breast Oncology Program, Dana-Farber Brigham Cancer Center, Boston, MA USA; 3 Harvard Medical School, Boston, MA USA; 4 Broad Institute of MIT and Harvard, Boston, MA USA

**Keywords:** Breast cancer, Metastasis

## Abstract

A significant subset of patients with metastatic breast cancer develops brain metastasis. As efficacy of systemic therapies has improved and patients live longer with metastatic breast cancer, the incidence of breast cancer brain metastases has increased. Brain metastases pose a clinical challenge in diagnosis, treatment, and monitoring across all breast cancer subtypes, and better tools are needed. Liquid biopsy, which enables minimally invasive sampling of a patient’s cancer, has the potential to shed light on intra-cranial tumor biology and to improve patient care by enabling therapy tailoring. Here we review current evidence for the clinical validity of liquid biopsy in patients with breast cancer brain metastases, with a focus on circulating tumor cells and circulating tumor DNA.

## Introduction

Central nervous system (CNS) metastases (including brain metastasis [BM] and leptomeningeal disease [LMD]) are the most common intracranial malignancy^1^. About one in five patients with metastatic breast cancer (MBC) will develop BM during their course of disease^1^. In autopsy studies, the prevalence is higher, reaching 40% of patients with lethal MBC^1^. Traditionally, BM were associated with very poor prognosis, however, novel therapies and treatment modalities are changing this paradigm in specific patient subsets. Patients with HER2-positive and triple-negative breast cancer subtypes have higher incidence of BM of up to 50% in some series with recent data suggesting an increase in the overall incidence of MBC-related BM (BCBM)^2^. This increase may be secondary to the efficacy of novel systemic therapies, specifically that of HER2-directed agents. LMD is a specific form of CNS metastasis, in which tumor cells infiltrate the leptomeninges, and associated with a dismal prognosis^3^. The clinical incidence of LMD is estimated to occur in up to 7% of breast cancer patients, enriched for patients with aggressive phenotypes (e.g., high grade, triple negative) and lobular histology^4^.

The pathophysiology of BM and LMD development is an area of active research. The mechanisms involved in tumor cells’ seeding are complex and involve multiple additional players, including the blood-brain barrier (BBB) and the microenvironment^1^. The BBB describes an insulating physiologic structure of microvasculature which closely regulates the passage of molecules and cells into and out of the brain microenvironment. The BBB is composed of endothelial cells, pericytes, astrocytes, and dual basement membrane. Metastasizing tumor cells are thought to reach the brain hematogenously, penetrate the BBB, and then colonize and proliferate in the brain tissue. Metastatic deposits then arise at the junction of white-gray matter and vascular watershed areas, where most BM are diagnosed in clinical practice^1,5^. In experimental models, brain penetration necessitates significantly longer periods when compared to other tissues, potentially explaining the preference towards slower-flowing vessels to enable more time for the metastasizing cells to complete their passage through the BBB^5^. Following metastasis formation, the BBB architecture is distorted and the insulating features are altered in a formation known as the blood-tumor barrier (BTB).

Recent studies have shown the presence of functional lymphatic vessels into the CNS^6^, but is unlikely that it has a role in BM development. Although the meningeal lymphatic system is involved in both brain tumor cells spreading into the external lymphatic system and in antitumor immune response^7^, this occurs through the lymphatic drainage from the CNS to the periphery. Given the absence of an inflow lymphatic system, this does not appear to be a possible route for metastatic tumor cells to penetrate the brain.

Studies have shown that an immune-suppressive environment develops in BM, further supporting tumor growth^1,5^. BCBM have a distinct set of features when compared to BM from other tumor types; more commonly there are multiple metastases, the interval between primary diagnosis and BM diagnosis is longer, and patients with BCBM are more often subsequently diagnosed with LMD as compared to other primary tumor types^8^.

The mutational landscape of BCBM is different from primary breast tumors or extracranial metastases. BCBM are enriched for alterations in specific pathways such PTEN loss and HER2 amplification^9,10^. Intriguingly, the genomic alterations in BCBM can be divergent from other metastases or from the primary tumors, highlighting the need to analyze brain lesions (or brain-derived circulating factors) in order to gain insights into their biology, and to identify targets for therapeutic interventions^11^.

**Fig. 1:**
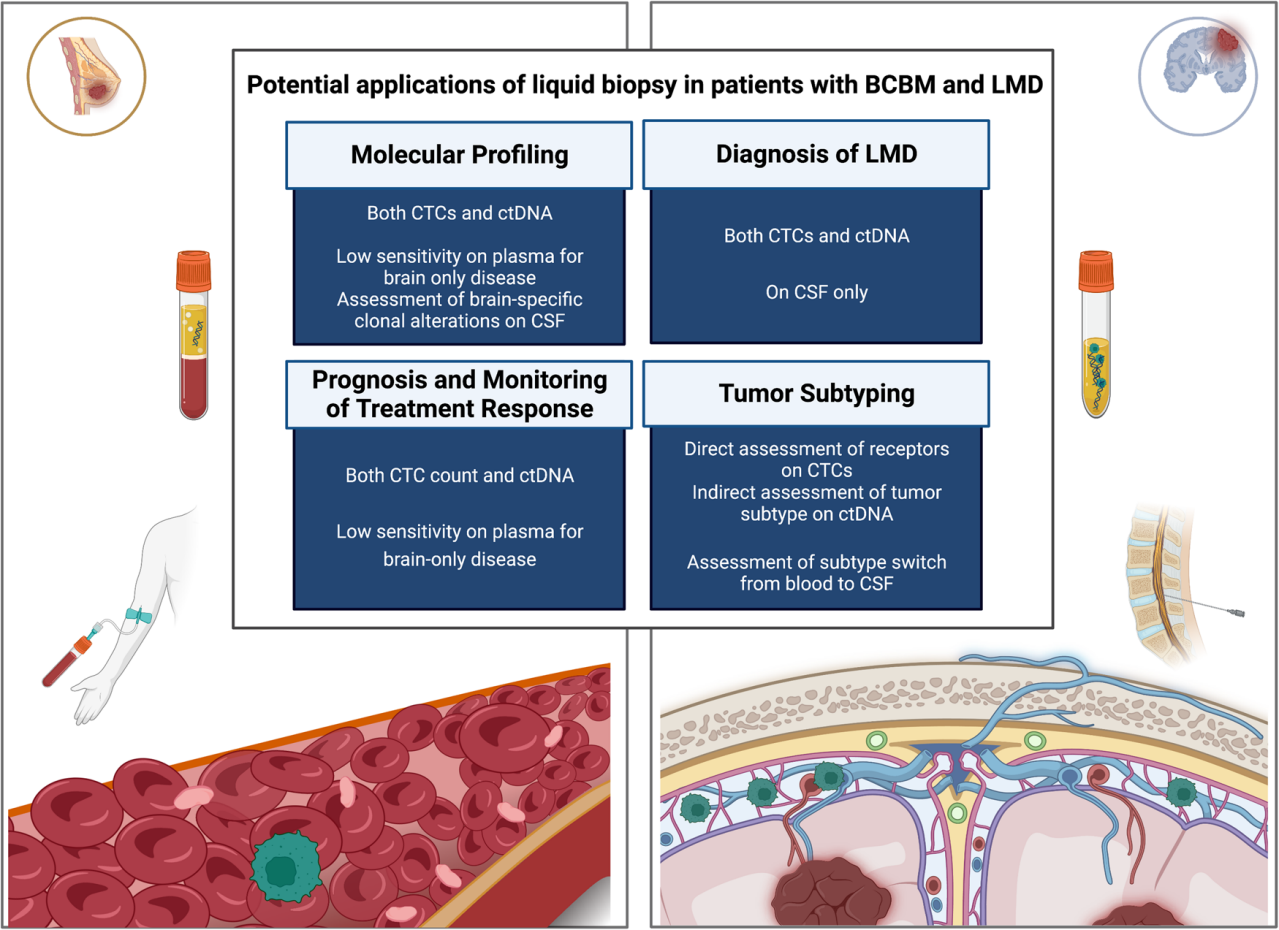
Potential applications of liquid biopsy in patients with BCBM and LMD. Created with BioRender.com. BCBM breast cancer brain metastasis, CSF cerebrospinal fluid, CTC circulating tumor cells, ctDNA circulating tumor DNA, LMD leptomeningeal disease.

Historically, only a limited number of systemically-administered cytotoxic agents were considered as having intracranial BBB-penetrant activity^12^. During the past two decades, with the rise of personalized medicine tools enabling tumor genotyping combined with the availability of targeted therapies (e.g., receptor tyrosine kinase inhibitors, PARP inhibitors), the clinical need to analyze BM for biomarkers has further evolved. Historically, analysis of CNS metastasis clinical samples began with material obtained from surgical specimens (e.g., craniotomy), and later on shifted to analysis of less-invasive sources, such as plasma or cerebrospinal fluid (CSF) from lumbar puncture. In the current manuscript, we will mainly review the contemporary data utilizing minimally-invasive analyses of BCBM-derived factors, mainly cell-free DNA (cfDNA) and circulating tumor cells (CTCs) (Fig. 1 and Table 1).

**Table 1:** Major studies investigating the role of CTCs and/or ctDNA in patients with BCBM/LMD.

Ref.	CTC\ctDNA	Plasma\CSF	Technology	Study aim	Conclusion(s)
Patel, A. S. et al. 2011^31^	CTC	CSF	CellSearch	Correlation between CSF CTC count and tumor response in patients with BCBM	CSF CTC count correlated with treatment response in patients with BCBM
Zhang, L. et al. 2013^18^^8^	CTC	plasma	Immuno-fluorescence with gene amplification by FISH	Molecular profiling of CTC	Identification of a brain metastasis selected markers proteomic signature from EpCAM-negative CTCs in patients with BCBM
Pierga, J. Y. et al. 2013^24^	CTC	plasma	CellSearch	Correlation between CTC clearance and treatment outcomes in patients with BCBM receiving first-line capecitabine plus lapatinib	CTC clearance is associated with improved CNS response and survival in patients with BCBM
Magbanua, M. J. M. et al. 2013^45^	CTC	CSF	Immunomagnetic enrichment and fluorescence-activated cell sorting	Molecular profiling of CTC	Isolated CTC can be successfully analyzed with NGS approaches
Boral, D. et al. 2017^16^	CTC	plasma	CellSearch, multi-parametric flow cytometry and DEPArray	Molecular profiling of CTC	BCBM-associated CTCs have a unique transcriptional signature
Riebensahm, C. et al. 2019^17^	CTC	plasma	CellSearch, Ficoll-based density centrifugation	Detection and molecular profiling of CTC	Detection of CTCs is an adverse prognostic factor in patients with BCBM.
Lin, X. et al. 2017^30^	CTC	CSF	CellSearch	Diagnosis of LMD	CTC is superior to standard CSF cytology in LMD diagnosis
Darlix, A. et al. 2022^27^	CTC	CSF	CellSearch		
Malani, R. et al. 2020^38^	CTC	CSF	CellSearch	CTC monitoring in patients with LMD receiving IT trastuzumab	CTC count is more sensitive than standard cytology in LMD diagnosis, and outperforms MRI and cytology in estimating response to IT therapy
Jacot, W. et al. 2019^48^	CTC	CSF	CellSearch	Identification and targeting of HER2-positive CNS CTCs in patients with HER2-negative MBC	HER2-directed therapy does not improve outcomes in patients with HER2-negative MBC and HER2-positive CNS CTCs
Parsons, H. A. et al. 2021^49^	CTC	CSF	CellSearch		
Diaz, M. et al. 2022^39^	CTC	CSF	CellSearch	Prognostic role of CTC in patients with newly diagnosed LMD	CTC count correlates with survival and outperforms imaging in patients with LMD
De Mattos-Arruda, L. et al. 2015^63^	ctDNA	Plasma, CSF	Targeted NGS panel	Molecular profiling of CSF ctDNA versus plasma ctDNA	Profiling of CSF ctDNA is more informative than plasma ctDNA in describing molecular alteration of BCBM. CSF ctDNA correlates with brain tumor burden. CSF ctDNA is superior to standard cytology for LMD diagnosis
Pentsova, E. I. et al. 2016^67^	ctDNA	CSF	Targeted NGS panel	Molecular profiling of ctDNA	CSF ctDNA profiling reflects genomic features of BCBM
Siravegna, G. et al. 2017^26^	ctDNA	plasma, CSF	ddPCR, WES	Correlation between ctDNA dynamics and treatment response	Case report; CSF ctDNA dynamics correlates with treatment response
Zhao, Y. et al. 2019^73^	ctDNA	CSF	Targeted NGS panel	LMD diagnosis	CSF ctDNA has better sensitivity than cytology or imaging for diagnosis of LMD
White, M. D. et al. 2021^70^	ctDNA	Plasma, CSF	Ultra-low-pass WGS	LMD diagnosis	CSF ctDNA improves LMD diagnostic accuracy over standard cytology
Angus, L. et al. 2021^71^	ctDNA	CSF	Targeted NGS panel, mFAST-SeqS	LMD diagnosis	CSF aneuploidy is a promising biomarker for LMD diagnosis
Fitzpatrick, A. et al. 2022^69^	ctDNA	Plasma, CSF	Ultra-low-pass WGS	LMD diagnosis and monitoring of treatment response	CSF ctDNA improves LMD diagnostic accuracy over standard cytology and imaging and correlates with treatment response.

*BCBM* breast cancer brain metastasis, *CSF* cerebrospinal fluid, *CTC* circulating tumor cells, *ctDNA* circulating tumor DNA, *ddPCR* droplet digital polymerase chain reaction, *FISH* fluorescence in situ hybridization, *IT* intra-thecal, *LMD* leptomeningeal disease, *MBC* metastatic breast cancer, *mFAST-SeqS* modified fast aneuploidy screening test-sequencing system, *MRI* magnetic resonance imaging, *NGS* next-generation sequencing, *WES* whole-exome sequencing, *WGS* whole-genome sequencing.

## Circulating tumor cells

CTCs are cells shed from the primary tumor or metastatic sites into the peripheral blood circulation. Overall, in patients with MBC not enriched for patients with BCBM, CTCs can be identified in up to 70% of cases using a 1 CTC/7.5 mL cut-off, and in up to 50% of cases using a 5 CTC/7.5 mL cut-off^13^. Various assays have been developed to detect CTCs in the bloodstream. First, CTCs are enriched based on biological or physical properties; second, immunological, molecular, or functional assays are used to identify CTCs among the surrounding white blood cells^14^. Among others, nuclear staining and expression of EpCAM and cytokeratins have been most frequently used to discriminate between CTCs from non-tumoral mesenchymal blood cells^14^. The CellSearch® assay, which is the only U.S. Food and Drug Administration (FDA)-approved method to detect CTCs in MBC, identifies CTCs based on the following criteria: EpCAM+, cytokeratin+, CD45−, DAPI+^15^.

Although the exact mechanism leading to development of BCBM is unknown, the extravasation of CTCs through the BBB into the CNS is likely to occur. However, how to identify and characterize these BM-initiating CTCs (BMICs) is under investigation. Interestingly, preclinical studies showed that mesenchymal-like, EpCAM-negative CTCs can generate BM^16–18^. This is of particular interest considering that most CTC detection methods target EpCAM-positive cells, and thus may miss BMICs. Therefore, alternate approaches may be more suitable for CTCs detection in patients with BCBM. To shed light on this question, Klotz and colleagues generated patient-derived CTC lines via ex-vivo cultures of CTCs isolated from patients with BCBM^19^. They observed that these cell lines can generate metastases when injected into immune-deficient mice, with a distribution pattern that reflects organ involvement in corresponding patients. Moreover, the authors identified Semaphorin 4D (SEMA4D) and MYC as key markers of BMICs, the former as mediator of BBB transmigration, and the latter as regulator for adaptation of CTCs in the brain microenvironment^19^.

In patients with MBC, enumeration of CTCs correlates with treatment response and survival^13^, whereas genomic and transcriptomic profiling of CTCs may provide information about tumor biology^20,21^. In patients with primary brain tumors or BM from solid tumors, prevalence of peripheral CTCs is relatively low, especially in case of brain-only disease due to the presence of the BBB^22–25^. Moreover, whether peripheral CTCs do reflect the biology of brain metastasis in patients with both intra- and extra-CNS disease is debated. Hence, ‘neurosystemic dissociation’ of response is not rare in patients with BCBM^26^. In addition, recent data indicate that CTCs can be detected in other non-blood sources, including CSF^25,27^. To date, CSF-CTCs proved to be a potentially helpful biomarker for diagnosis of LMD, an assessment treatment response and prognosis, and as a source of genomic information for tumor subtyping and identification of actionable alterations.

### Diagnosis of LMD via CTCs

Traditionally, a diagnosis of LMD relies on a combination of clinical signs and symptoms, radiographic findings, and conventional cytology of CSF samples^28^. Albeit the gold standard, CSF cytology has a relatively low sensitivity, estimated as 44–67% with a single assessment, and up to 84–91% with repeated sampling^29^. In addition to low abundance of tumor cells in the CSF, this technique may be limited by technical and analytical challenges. Sampling via lumbar puncture requires expertise, CSF sample volume has to be at least 3 mL, and analysis must be performed rapidly given fast clearance of tumor cells after sampling^27^. In case of negative cytology, LMD may be prompted by symptoms and typical findings at brain and/or spine MRI, such as leptomeningeal contrast enhancement, cranial nerve enlargement, ventriculitis, and/or non-obstructive hydrocephalus^28^. Thus patients may be diagnosed with LMD even in the absence of a positive CSF cytology.

In order to increase sensitivity of LMD diagnosis, several studies investigated the prevalence of CTCs in CSF and its correlation with cytology, repurposing assays initially validated for peripheral CTCs^27,29–31^. To date, the use of CSF-CTCs for LMD diagnosis reached a significantly higher sensitivity than cytology at first lumbar puncture (78–100% vs 44–67%), and a specificity of 84–100%^29^. In contrast to conventional cytology, which relies on standard staining and immunohistochemistry for cytokeratin (CK) and/or epithelial membrane antigen (EMA), CTC assays enrich for epithelial tumor cells by more sensitive methods. The CellSearch system includes anti-EpCAM antibodies conjugated with ferromagnetic particles in addition to fluorescently conjugated antibodies^15^, whereas others are based on multiple fluorescently conjugated antibodies targeting a variety of cell surface markers that are subsequently enumerated by flow cytometry^29^.

However, none of these studies proved the clinical utility of CTCs for patients with MBC. The small sample size, inclusion of many tumor subtypes and non-randomized design of these studies are major limitations. Moreover, the interpretation of false-positive results in these studies is challenging. As the ground truth was represented by cytology in most of these studies, false-positives may represent true-positives missed by false-negative cytology. Or false-positives may represent excessive sensitivity of CTC-based assays for what clinically may or may not behave as classic LMD and require LMD-focused therapies. Hence, the optimal cut-off for diagnosis has not been defined. Considering the evidence generated so far, the Response Assessment in Neuro-Oncology (RANO) Leptomeningeal Metastasis and the RANO Brain Metastasis Working Groups recommend considering CTCs as tools for high-sensitivity detection, but presence/absence of malignancy should be confirmed by formal cytology^29^.

### Prognostic value of CTCs

In 2004, the prognostic significance of plasma CTCs in MBC was first reported^32^. In this seminal study, CTCs were assessed in 177 patients both before starting a new line of therapy and at the first follow-up visit. At both time points, patients with a CTC count ≥5/7.5 mL had a significantly shorter progression-free and overall survival. Of note, CTC level was the most significant independent predictors of both endpoints, and these results led to the FDA approval of CellSearch as a prognostic assay in MBC.

Although several studies further confirmed the prognostic role of CTCs in this setting^13^, CTC count failed to show a meaningful predictive value. In two prospective, randomized, phase III studies, serial CTC monitoring and early treatment switching based on CTC count failed to improve long-term outcomes^33,34^. Hence, major international guidelines do not recommend routine use of CTCs to monitor response to therapy among patients with MBC^35,36^.

In patients with BCBM, peripheral CTC count similarly correlates with worse prognosis. The phase III LANDSCAPE trial assessed the efficacy of capecitabine plus lapatinib in patients with HER2-positive MBC and newly diagnosed, untreated brain metastasis. As a preplanned secondary objective, the prognostic role of CTCs at baseline and day 21 was investigated. Using a cut-off of 1 CTC/7.5 mL, prevalence of CTC-positive was 49% (20/41 patients) at baseline and 18% (7/38) at day 21, with CTC clearance observed in 11 patients. Despite the small sample size, significantly better outcomes were observed in patients with early CTC clearance than in CTC-positive patients at day 21, in terms of both CNS response (80% vs 29%, *p* = 0.01) and 1-year overall survival (83.9% versus 42.9%, *p* = 0.02)^24^. Similarly, Riebensahm and colleagues observed a significant association between poor survival after BCBM diagnosis and the presence of peripheral CTCs^17^. Interestingly, dynamics of peripheral CTCs appears to also correlate with response to brain radiation, with evidence of persistent vital CTCs in poor responders versus an increase in apoptotic CTCs in patients with local response to therapy^37^.

Although only a few small studies assessed CTCs in CSF and their correlation with outcomes in patients with BCBM and/or LMD, a trend towards high CTC count and short survival, and a correlation between decline in CTC count and response to intrathecal therapy was observed^31,38^. In patients with LMD, the prognostic value of CSF-CTCs was recently confirmed by a large retrospective analysis. In this study, in which 35 out of 101 patients included had MBC, risk of death was more than double in patients with a CSF-CTC count at or above the optimal cutoff of 61 CSF-CTCs/3 mL (hazard ratio 2.84, *p* = 0.002)^39^. More prospective studies are needed to understand whether CTCs in CSF can be routinely used to assess prognosis and guide therapy.

### Molecular profiling and tumor subtyping via CTCs

Breast cancer is highly heterogeneous, with diverse subclones that evolve under selective pressure. Although breast tumors are usually classified as either hormone receptor (HR)-positive/HER2-negative, HER2-positive, or triple-negative based on expression of receptors at diagnosis, the emergence of clones with phenotypically distinct characteristics may occur. Hence, a subtype switch, defined as either receptor gain or loss, is not rarely observed^40^. This phenomenon appears to be particularly frequent in patients with BCBM^41,42^ and has been ascribed to selective tropism of BM-initiating clones. As mentioned, HER2-positive BCBM clones have a high tropism for the brain microenvironment^43^.

As all other “cytology” specimens, CTCs can be characterized at the protein, RNA, and genome levels.

DNA sequencing of CSF-CTCs and matched primary tissue in patients with LM showed that although some alterations are shared, others can be identified in the CTCs only^44,45^. This finding reflects what was previously shown in large studies comparing mutational profiles of BM and primary tissues, and suggests the subclonal origin and branch evolution of BM^11^. Similarly, profiling of peripheral CTCs and matched tumor tissue in 3 patients with BCBM showed chromosomal aberrations with a high genomic clonality and mutations in pathways previously associated with BM including the Notch and PI3K pathways, cell cycle regulations, epithelial-mesenchymal transition, and chromatin remodeling^17^. Furthermore, transcriptomic analysis of CTCs allows for investigation of gene expression profiles. For example, via whole-genome mRNA microarray of peripheral CTCs, Boral and colleagues described a unique “CTCs gene signature” that was distinct from primary breast cancer tissues^16^. By comparing staining and transcriptional profiling of CTCs between patients with and without BM, the authors also identified that patients with BCBM have more CTCs with high ki67 staining, and a 126-gene signature that was significantly altered between the two groups, with higher Notch signaling, hyperactivation of pro-inflammatory and immunomodulatory networks among patients with BCBM^16^.

Finally, CTC analysis allows the assessment of protein expression. Given its therapeutic role, most studies have focused on HER2 expression by CTCs. In line with the subtype switch observed in tissue analyses, many cases of HER2 gain have been observed across different series and reported in up to 40.6% of cases^27,46,47^. Hence, HER2-positive CTCs may reflect clonal HER2 heterogeneity eventually missed by tissue analyses due to sampling bias, and thus be used as a predictive biomarker of benefit from anti-HER2 therapy. Two clinical trials that investigated the efficacy of anti-HER2 therapy in patients with HER2-negative MBC and HER2-positive peripheral CTCs failed to demonstrate a convincing benefit (response rate of 5% with trastuzumab-navelbine; 9.1% with trastuzumab emtansine)^48,49^. However, whether HER2 assessment on CSF-CTCs would be effective to detect and target HER2 gain in patients with BM is unknown. For instance, HER2 staining on CTCs may be helpful to identify a subgroup of tumors with HER2 score 0 at immunohistochemistry who may derive benefit from trastuzumab deruxtecan, an anti-HER2 antibody-drug conjugate with high brain penetration which is currently approved only for HER2-positive and HER2-low breast cancer^50,51^.

Taken together, CSF-CTCs might shed light on intracranial disease dynamics and enable studying its features, but further work is needed to decipher the clinical role of these cells in patient care. In summary, CTCs are promising biomarkers in patients with BCBM and/or LMD and may help to profile the unique molecular landscape of brain lesions, to confirm LMD diagnosis, and to monitor for treatment response. However, evidence proving their clinical utility is still lacking and prospective trials are warranted.

## Circulating tumor DNA

The term cfDNA refers to double-stranded DNA fragments bound to histones in circulation that can be detected in blood and other body fluids. Although most plasma cfDNA is derived from white blood cells, in patients with cancer a proportion of cfDNA consists of circulating tumor DNA (ctDNA) released by tumor cells via secretion or following apoptosis or necrosis^52^. The fraction of cfDNA composed by ctDNA is highly variable according to tumor burden and histology, host factors, and type of fluid sampled^52^. Moreover, sensitivity of ctDNA assays is highly variable and depends on the analyzed volume, ranging from 1 × 10^−6^ of cutting-edge minimal residual disease assays to 0.1% of commercially available next-generation sequencing panels^53–55^. With modern assays, ctDNA can be identified in almost all patients with MBC, given the relatively high amount of circulating DNA and genomic aberrations^56^.

The amount of plasma ctDNA correlates with tumor burden in patients with MBC. Hence, monitoring ctDNA dynamics has been shown to correlate with treatment response^57–60^. However, discordancy between CT scan results and ctDNA dynamics has also been observed^61^, and its clinical utility still has to be proven^14^. Moreover, ctDNA analysis can identify tumor alterations that may help in treatment choice, both as predictor of response mutations (e.g., PIK3CA mutations for PIK3CA-inhibitors) and resistance (e.g., ESR1 mutations).

However, similar to CTCs, sensitivity of ctDNA assays is much lower in patients with brain-only metastasis^62–65^. Hence, CSF may represent an alternative source for isolation of ctDNA. Although potential applications of ctDNA and CTCs are similar for patients with BCBM, the two techniques are different and not completely overlapping, and might be considered complementary.

### Diagnosis of LMD via ctDNA

The presence of BTB and the direct contact between intracranial tumors and CSF make it an excellent source of ctDNA. Moreover, the CSF contains fewer white blood cells, thus ctDNA assays have increased sensitivity for low variant allele fraction (VAF) variants due to the reduced noise caused by non-tumor cfDNA^66^.

Isolation of ctDNA from CSF has been investigated as an alternative approach of LMD diagnosis. Although most of the evidence available relies on retrospective studies including few patients across different tumor types, in all cases ctDNA outperformed cytology in LMD diagnosis^63,67^. Hypothetically, ctDNA could even anticipate LMD diagnosis, as it has been detected in one patient without any signs or symptom of LMD, but with LMD diagnosed at autopsy^63^.

In addition to traditional ctDNA targeted DNA sequencing assays, a few studies have investigated ultra-low pass whole-genome sequencing (ULP-WGS) on CSF-ctDNA for LMD diagnosis. ULP-WGS is a relatively low-cost and rapid tool that relies on low coverage (0.1x) WGS and, differently to targeted approaches, allows to estimate tumor content in ctDNA without prior knowledge of the mutational profile. Moreover, it is optimized to achieve high sensitivity even in low volume samples^68^. In a series of 30 cases (24 LMD-positive and 6 LMD-negative cases), ctDNA fraction in CSF was found to be significantly higher in patients with LMD, compared to patients without intracranial involvement (median 0.57 vs 0.03; *p* < 0.0001) using ULP-WGS^69^. Considering a ctDNA fraction cut-off of 0.10, all patients with LMD were ctDNA-positive whereas all patients without LMD were ctDNA-negative. In line with prior reports, ctDNA fraction was significantly lower in plasma than CSF in LMD cases, and 12/22 samples were below the detection level^69^. A similar analysis on 30 patients across different tumor types reported an accuracy of 94% (sensitivity 93%, specificity 100%) to detect LMD. Of note, 5 patients with BM abutting the CSF were ctDNA-positive despite the absence of radiographic, symptomatic, or cytologic evidence of LMD, suggesting the limitations of ctDNA to diagnose LMD in the setting of BCBM, or the enhanced sensitivity to detect sub-clinical disease^70^.

As an alternative to ULP-WGS, an equally fast and affordable technique to assess ctDNA fraction is the modified fast aneuploidy screening test-sequencing system (mFAST-SeqS) method, which employs selective amplification of long interspaced nuclear elements (LINE-1 sequences) that are sparce throughout the genome. In a series of 121 patients, the mFAST-SeqS had a false-negative rate of 23.1% and a false-positive rate of 5.4%. Of note, 4/14 cytology-negative, aneuploidy-positive patients further developed LMD. Despite the low sample size, which does not allow for determination of the best cut-off in this setting, these data demonstrated the feasibility of mFAST-SeqS for LMD diagnosis and higher sensitivity when compared to cytology alone^71^.

### Molecular profiling of ctDNA

ctDNA profiling via whole exome or targeted sequencing allows not only for quantification of tumor fraction, but also for identification of molecular alterations with prognostic and/or predictive value.

In a pivotal study, De Mattos-Arruda and colleagues compared the mutational profile of CSF and plasma ctDNA in patients with primary brain tumors or BM^63^. They found CSF ctDNA to be representative of brain tumors, as identified alterations were confirmed in brain tumor tissue samples. Furthermore, CSF-ctDNA was more informative than plasma ctDNA; although VAF of gene alterations in the CSF and plasma were comparable in patients with abundant visceral burden, in patients with brain-only disease the allele fractions in CSF ctDNA were significantly higher than in plasma (ctDNA found in 58% of cases vs. 0%, respectively). The authors also observed how CSF genotyping allows detection of several gene mutations absent from plasma ctDNA. Of note, some of the identified alterations in CSF ctDNA were specific of the brain metastasis and not detected in extracranial lesions^63^. Hence, sequencing of CSF ctDNA may reliably provide the mutational profile of brain lesions in most patients with BM or primary brain tumors. This is of particular interest given the potential clinically relevant value of liquid biopsy to genotype BCBM, in light of the low sensitivity of plasma ctDNA in patients with brain-only disease (or ‘neurosystemic dissociation’). CSF profiling has been shown to identify both candidate mechanisms of resistance and potentially druggable alterations in patients with BCBM, such as PIK3CA mutations or PTEN loss^26,67,72,73^.

Furthermore, novel computational analyses of cfDNA are now exploring fragmentation patterns to reveal the occupancy of nucleosomes in cells-of-origin^74^. This structural study, called nucleosome profiling, has been primarily used for cancer detection and prediction of tumor tissue-of-origin^75^. However, profiling of nucleosome accessibility and transcriptional regulation may be also used to infer transcriptional profiles. In patients with breast cancer, the Griffin framework proved to properly perform estrogen receptor (ER) subtyping from ULP-WGS of ctDNA and discriminate between ER-positive and ER-negative tumors^76^. Given the frequency of ER loss and HER2 gain in BCBM, the availability of a CSF-based assay to assess receptor status of BCBM\LMD would allow to bypass the current need for invasive procedures^41,77^. Although the clinical utility of these technologies still must be proven, this would be of great interest to better target the dynamic evolution of CNS metastasis over time.

### ctDNA as surrogate biomarker of response

ctDNA dynamics in plasma correlates with treatment response in patients with MBC^78–80^. In a proof-of-concept study including 30 women with MBC, Dawson and colleagues proved that ctDNA dynamics is more sensitive than traditional tumor markers (CA 15-3) and CTCs in predicting response and progression^59^. Of note, most patients in this study were tested using an “old”, tumor-informed ctDNA assay tracking only PIK3CA and TP53 alterations via digital PCR or tagged-amplicon deep sequencing, as the whole-genome sequencing approach was used only for 9 patients. Hence, only 30 out of the 52 patients initially screened had tumor alterations identified on the primary tissue allowing for informed ctDNA tracking. Modern techniques based on tissue whole-exome or -genome sequencing allow to track multiple mutations, thus increasing sensitivity significantly even in tumor with low mutational burden.

More recent analyses from different patient cohorts confirmed the role of ctDNA in predicting response and disease progression^57,80^, although its clinical utility still has to be proven^14^. In patients with BCBM, CSF ctDNA levels similarly showed to correlate with disease burden, treatment response, and prognosis^63,69–71^.

Interestingly, tumor-informed approaches showed to be more sensitive in CSF than plasma. Hence, only ctDNA VAF in CSF, and not in plasma, was shown to decrease with surgical resection and/or responses to systemic therapy and increase with tumor progression in a small cohort of patients with minimal or no extracranial disease^63^.

Tumor-agnostic methods such as ULP-WGS and mFAST-SeqS have been also applied for sequential monitoring of CSF ctDNA, and both showed to properly correlate with treatment response and survival^69–71^. In particular, ctDNA suppression during intrathecal treatment was shown to correlate with both response to therapy and survival^69^. Of note, ctDNA levels were found to significantly increase after surgery and radiation therapy, stressing the need to account for confounding factors to properly interpret ctDNA dynamics^70^.

Altogether, ctDNA (especially from CSF) is a promising approach to study and follow-up for intracranial metastatic lesions. However, its clinical application is still limited by the invasiveness of CSF sampling, which requires either repeated lumbar punctures or placement of an Ommaya catheter, and by the lack of robust clinical data proving its clinical utility.

## Other approaches

In addition to ctDNA and CTCs, patients with BCBM were found to have distinctive profiles of various molecules, both in blood and CSF, compared to patients without BCBM. For example, patients with BCBM have higher plasma levels of specific long non-coding RNA^81^, specific microRNAs^82^, and proteins such as lactate dehydrogenase-A^83^, Tau^84^, and Angiopoietin-like 4^85^. Intriguingly, lower quantity of plasma exosomes with an increased protein content were found to be associated with the presence of BCBM^86^. In an elegant mouse model, the complement protein C3 was found to be secreted by intracranial breast cancer cells to increase the permeability of the BBB and facilitate the entry of factors to support additional tumor growth^87,88^. Furthermore, based on differential metabolic adaptations between BCBM and extracranial metastases^89^, analysis of CSF metabolites also demonstrated distinct features in patients with BCBM, e.g., lower alanine and lactic acid levels^90^.

Unfortunately, despite a plethora of preclinical evidence involving non-cfDNA biomarkers, almost all the reports remain anecdotal without translation into clinical studies. We believe that one of the major challenges to promote this field is the relative unavailability of CSF and brain metastases tissues. Therefore, more investment in the establishment of biobanks is desirable to facilitate better and more robust biomarkers research.

## Future perspectives

Liquid biopsy for studying CNS lesions is largely challenged by anatomic constraints. Analysis of CSF is technically simpler than plasma, but requires invasive procedures (e.g., lumbar puncture) to obtain it. The most convenient liquid biopsy approach for BCBM and/or LMD would involve blood, but the limited available data suggest that CSF ctDNA analysis may best reflect CNS disease, whether as a diagnostic test or for tumor genotyping. Of course, the utility of CSF ctDNA analysis would ideally be demonstrated in a prospective cohort with a robust and standardized assay. Importantly, the relatively low prevalence of BCBM\LMD and the challenges in access to CSF means that practically speaking, multi-center collaborations using robust and cutting-edge assays will be required to generate answers to the numerous questions in this field. In addition, we recommend additional research with chromatin and methylation-focused liquid biopsy assays in the setting of CNS metastasis. Chromatin and methylation analyses might shed light on tumor cells’ gene expression or even identify damage to neighboring healthy tissues as a biomarker for a growing metastasis^75,76,91^.

**Fig. 2:**
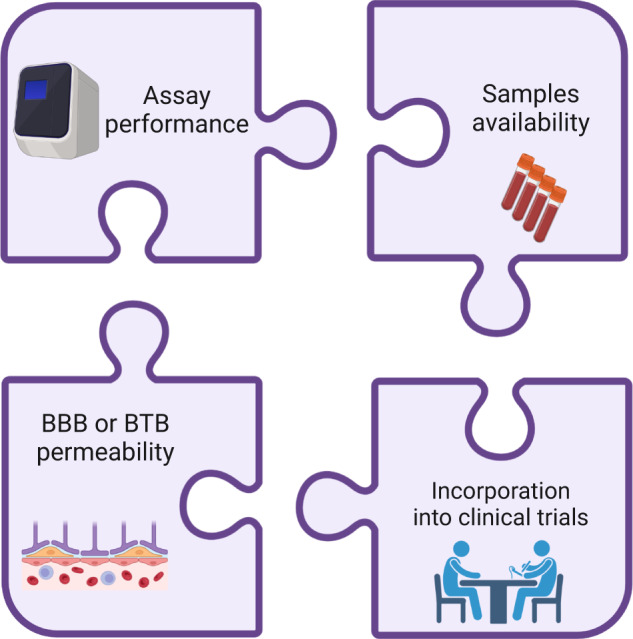
Challenges for liquid biopsy development in patients with central nervous system metastasis from breast cancer. Created with BioRender.com. BBB blood-brain barrier, BTB blood-tumor barrier.

Overall, we believe that past and future studies in this field are limited due to several key barriers (Fig. 2), which must be addressed in order to make a significant progress. First, to fulfill the vision of blood-based liquid biopsy, assay performance should be considerably optimized to allow detection of brain-derived ctDNA in the plasma of patients with BCBM, e.g., the development of ultra-sensitive tumor-uninformed assays can be a potential solution to this challenge. Second, for technical reasons, it may be that CSF-based assays will always outperform blood-based assays, and there is dramatic room for improvement in our current standard panel of conventional cytology, cell counts, glucose, and total protein. To do this will require prospective studies to demonstrate the impact and value of ctDNA-based, CTC-based, or other novel assays in the care of patients with BCBM and/or LMD. Third, multi-institutional collaboration and resource investment in the creation of BM and LMD biobanks, with clinical data, linked to tumor, CSF, and plasma samples will significantly facilitate translational research efforts in this field. Fourth, BBB\BTB permeability is one of the main obstacles for detecting plasma ctDNA which represents intra-cranial lesions. Understanding the currently unknown mechanisms of ctDNA transport through the BBB is an important step for improving systemic detection of brain-derived ctDNA. Lastly, liquid biopsy use should be incorporated into clinical trials involving patients with BCBM and LMD. Such trials will also facilitate the collection of precious bio-specimens for future analysis and set the stage for testing various assays with a true potential to enter clinical routine. Education and outreach to both health care professionals and patients regarding the importance and potential future impact of CSF collection for research purposes, despite the obvious barriers, are critical to the success of such biobanking efforts.

In conclusion, liquid biopsy in patients with BCBM has a great potential to impact clinical care. Further research focused on the current barriers is clearly required to overcome the biological and technical challenges ahead.

## Data Availability

Data sharing is not applicable to this article as no datasets were generated or analyzed during the current study.
